# Epinecidin-1, a highly potent marine antimicrobial peptide with anticancer and immunomodulatory activities

**DOI:** 10.1186/s40360-019-0309-7

**Published:** 2019-05-28

**Authors:** Alireza Neshani, Hosna Zare, Mohammad Reza Akbari Eidgahi, Azad Khaledi, Kiarash Ghazvini

**Affiliations:** 1 Antimicrobial Resistance Research Center, Mashhad University of Medical Sciences, Mashhad, Iran; 2 Department of Microbiology and Virology, Faculty of Medicine, Mashhad University of Medical Sciences, Mashhad, Iran; 3 Student Research Committee, Mashhad University of Medical Sciences, Mashhad, Iran; 4 Biotechnology Research Center, Semnan University of Medical Sciences, Semnan, Iran; 5 Infectious Diseases Research Center, Department of Microbiology and Immunology, Faculty of Medicine, Kashan University of Medical Sciences, Kashan, Iran

**Keywords:** Antimicrobial peptide, Epinecidin-1, *Epinephelus coioides*, Anticancer, Immune response

## Abstract

**Background:**

Antibiotic-resistant pathogens are an emerging threat in this century. Epinecidin-1 is a multi-functional Antimicrobial Peptide (AMP) produced by Orange-spotted grouper (*Epinephelus coioides*) has been shown to have extensive potentials as an alternative for current antibiotics. Due to the huge costs for the study and the production of a new drug, if an antimicrobial peptide has other beneficial functions in addition to antimicrobial activities, it would be preferred.

**Methods:**

In this study, properties and applications of Epinecidin-1 were investigated and addressed comprehensively. To achieve this, the Google Scholar search engine and three databases of PubMed, Scopus, and Web of Science were used.

**Results:**

Epinecidin-1 is a cationic AMP with an alpha-helical structure. Seven functional usages of this peptide have been reported in the literature including antibacterial, antifungal, antiviral, antiprotozoal, anticancer, immunomodulatory, and wound healing properties. Moreover, this peptide has high potential to be used as an active ingredient in cleaning solutions as well as application in vaccine production.

**Conclusion:**

Due to significant antimicrobial activities tested on bacteria such as *Staphylococcus aureus* and *Helicobacter pylori* and also wound healing properties, Epi-1 has high potential to be considered as an important candidate for the production of new drugs and treatment of various infections including diabetic foot ulcer and peptic ulcer. Moreover, adjuvant-like properties of Epi-1 make it a suitable candidate for the studies related to an adjuvant. Other attractive properties such as anticancer effects have also been reported for this peptide which encourages further studies on this peptide.

## Background

The emergence of antibiotic-resistant pathogens is one of the main concerns of health organizations in recent years. Various types of drug resistance are rapidly being developed for the pathogens while were previously treatable using simple antibiotics. However, the introduction process of new antibiotics is greatly slow. Evidence shows that the risk of post-antibiotic era is relatively close to humans [1, 2]. Therefore, finding appropriate alternatives is highly required.

Antimicrobial Peptides (AMPs) are one of the most potent alternatives, and many studies have been conducted for them in recent years. They usually have a short length (less than 50 amino acids), and cell membrane attack is the most known mechanism of their antimicrobial activity. AMPs are often extracted from a living organism, or they are peptide derivatives [3]. With the improvement of bioinformatics science in recent years, synthetic AMPs have also been designed and produced. One of the main advantages of these AMPs is their good antimicrobial effect against multidrug-resistant pathogens. Although these peptides are usually known by their antimicrobial effect, they have some other properties including anti-cancer, immunomodulatory, wound healing, and antioxidant effects [3–5].

**Fig. 1:**
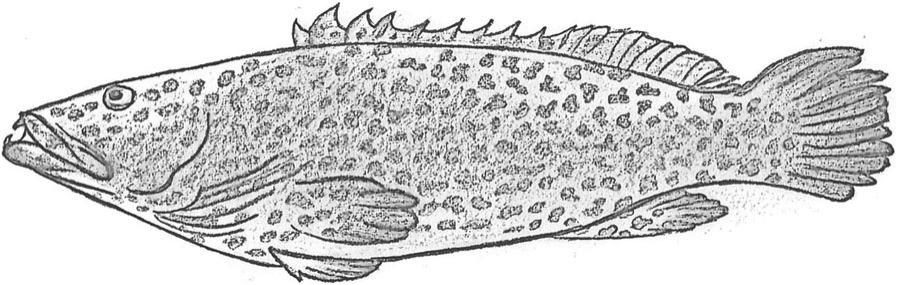
Orange-spotted grouper (*Epinephelus coioides*)

Aquatic animals are known as a rich source of AMPs as a part of their innate immunity, and various types of AMPs have been identified in these organisms [6]. Epinecidin-1 which is a naturally occurring peptide by orange-spotted grouper (*Epinephelus coioides*) (Fig. 1) has been identified since 15 years ago [7]. *E. coioides* is one of the common food sources for human especially in Southeast Asian countries [8].

Up to now, many studies have been performed to identify the Epinecidin-1 and mechanism of action. In this review, all studies related to this AMP are investigated with the aim of introducing various classified aspects of this peptide.

## Methods

### Search strategy

The search was performed in two steps. First, all English studies including the Epinecidin-1 AMP (up to March 25, 2019) were selected from PubMed, Scopus, Google Scholar, and Web of science databases. Only those original studies were included in our study that helped to clarify one of the Epinecidin-1 aspects, at least in one part or the whole of their research.

The search formula (Epinecidin-1[All Fields] OR Epinecidin-1[All Fields]) AND antimicrobial peptide[All Fields] was used for PubMed. The search formulas for Scopus and Web of science were similar to the PubMed search formula. The keywords of Epinecidin-1, Epi-1, Epinecidin-1 antimicrobial peptide and Epi-1 antimicrobial peptide were used for the Google Scholar search. In addition, the cited studies to our selected articles and references were reviewed and studies that could provide possible additional information on the structure, mechanism, and source of Epinecidin-1 were selected.

## Results

### 1. Epinecidin-1

Yin et al. in a study in 2003 made an orange-spotted grouper leukocyte cDNA library and sequenced more than 300 expressed sequence tags (ESTs) of cDNA [9]. Three years later, the evaluation and comparison of these nucleotide sequences with the present AMP sequences of GenBank database led to the identification of new AMP which named Epinecidin-1 due to the extraction from *E. coioides*. cDNA of Epinecidin-1 has 518 bp length (GenBank: AY294407.1), and its largest open reading frame is 204 bp. Epi-1 is initially synthesized as a 67 amino acids prepropeptide containing three domains of the signal peptide, mature peptide, and a prodomain. The Epi-1 gene consisted of three introns and four exons. Determination of domains and the number of amino acids have been performed by bioinformatics software, as the first 22 amino acids were considered as a signal peptide and the next 25 amino acids as the mature peptide [7]. In 2007, Pan et al. considered the first 21 amino acids as the signal peptide and the next 30 amino acids as the mature peptide. The amino acid sequence of Epinecidin-1 which introduced as a partial or full sequence by Yin et al. and Pan et al. are provided in Table 1. The third sequence of Table 1 was considered in the subsequent studies in the following years, due to the lower size and production cost. Investigation of various organs of *E. coioides* showed that the most expression of this AMP was related to the head, kidneys, intestines, and skin. While the immunohistochemical analysis showed that the peptide was localized in the gills and intestines [10]. This can be explained by the fact that the expression of Epinecidin-1 is different in main organs in the pathologic condition. Investigation of Epinecidin-1 gene expression in the *Vibrio harveyi*-infected Grouper has shown that expression of this peptide upregulated by the onset of infection and persisted for ten days. Interestingly, the highest expression occurred in the skin followed by gills, liver, and kidney [11].

**Table 1:** Epinecidin-1 derivatives and their characteristics

Study	Epi-1 derivative peptide sequence	Location in Epi-1 prepropeptide	pI [13]	Charge [13]	MW [13]
Yin et al. [7]	FIFHIIKGLFHAGKMIHGLVTRRRH	23–47	12.811	+ 5	2985.64134
Pan et al. [10]	GFIFHIIKGLFHAGKMIHGLVTRRRHGVEE	22–51	11.426	+ 3	3457.10874
	GFIFHIIKGLFHAGKMIHGLV	22–42	10.591	+ 2	2335.88454

Investigation of 1182 bp upstream of the Epinecidin-1 gene showed that there were some putative regulatory elements and binding motifs for transcription factors in this region. These sequences included TATA box (nucleotide − 34), three binding sites for CAAT enhancer-binding protein β (C/EBPβ) (nucleotide − 240, − 554, − 735), and two binding sites for hepatocyte nuclear factor (HNF) 1 (nucleotide − 667) and HNF-3b (nucleotide − 951). It has also shown that the Epinecidin-1 production in *E. coioides* is increased in exposure to the Lipopolysaccharide (LPS) and Polyinosinic:polycytidylic acid (poly(I):poly(C)) in a dose-dependent manner [7, 10, 12].

### 2. Structure and stability

Comparison of Epinecidin-1_23-47_ with other AMPs isolated from fish has shown that this peptide has a 71.4–78.6% identity with three peptides of Chrysophsin 1, 2, 3. After chrysophsins, the most identity was related to Moronecidin (69%), Pleurocidin WF3 (61.9%), and Piscidin3 (54.5%), respectively [7, 14, 15].

**Fig. 2:**
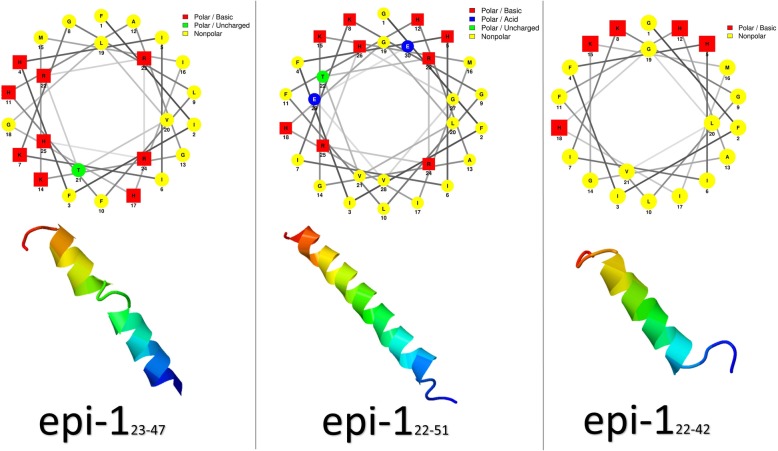
The spatial organization of Epinecidin-1 derivatives. Helical wheel diagrams (up) [7, 16], three-dimensional structure (down) [17]

Three derivatives of Epinecidin-1 have a molecular weight of 2.3–2.9 kDa and are highly cationic (Table 1). Based on the Schiffer-Edmundson helical wheel diagram, all three derivatives of this peptide have a secondary amphipathic α-helix conformation, and polar and non-polar amino acids are located in two different sides of structure [7, 16]. Epinecidin-1 has no disulfide bonds and its alpha-helical structure has been shown well by the three-dimensional structure provided by protein structure prediction software (Fig. 2) [17].

The stability of Epinecidin-1_22-42_ antimicrobial effect was also evaluated under different conditions. Exposure to Gamma irradiation reduced the antimicrobial effect; the minimum inhibitory concentration (MIC) was 39 μg/ml compared to the 9.7 μg/ml of the unirradiated control after exposing to methicillin-resistant *Staphylococcus aureus* (MRSA). Temperature rise also reduced the antimicrobial activity. The MIC was 312 μg/ml in exposure to MRSA after 5-min pretreatment at 40 °C compared to the control group which was 9.7 μg/ml at 25 °C. While temperature and irradiation showed a negative correlation to antimicrobial activity, acidic conditions had no significant effect on its activity. The highest activity of Epinecidin-1 was found under acidic conditions [18].

### 3. Antibacterial, antifungal, and antiprotozoal effects

AMPs usually have extended-spectrum effects [19]. So far, Epinecidin-1 antimicrobial activity has been evaluated and proved on 24 bacterial, six fungal, and one protozoan strains (Table 2). The MIC value of this peptide is quite competitive with antibiotics in exposure to many pathogens, and it can also kill antibiotic-resistant strains. The MIC values of three Epinecidin-1 derivatives in exposure to several pathogens are provided in Table 2.

**Table 2:** MIC values of three Epinecidin-1 derivatives in exposure to various pathogens

		MIC (μg/ml)		
		Epinecidin-1 _23–47_ [7]	Epinecidin-1 _22–51_ [10]	Epinecidin-1 _22–42_ [10, 18, 20, 28, 29, 31]
Gram-positive bacteria	*Listeria monocytogenes* (BCRC14930)	–	25	50
	*Bacillus subtilis*	> 200	–	–
	*Micrococcus luteus* (BCRC11034)	–	3.125	25
	*Staphylococcus aureus* (BCRC10780)	–	6.25	9.7–12.5
	*Streptococcus pyogenes* (BCRC10797)	–	25	25
	*Streptococcus agalactiae* (819)	–	12.5	50
	*Staphylococcus epidermidis* (BCRC10783)	–	12.5	> 100
	*Staphylococcus sp.* (BCRC10451)	–	6.25	50
	*Staphylococcus xylosus* (BCRC12930)	–	> 100	50
	*Streptococcus pneumoniae* (BCRC10794)	–	12.5	25
	*Staphylococcus aureus subsp.* (BCRC10782)	–	6.25	6.25
	*Propionibacterium acnes*	–	–	200
Gram-negative bacteria	*Helicobacter pylori*	–	–	8–12
	*Enterobacter aerogenes* (BCRC10370)	–	25	50
	*Enterobacter cloacae subsp.* (BCRC10401)	–	100	100
	*Vibrio alginolyticus*	–	12.5	100
	*Klebsiella oxytoca* (BCRC13985)	–	25	50
	*Salinivibrio costicola subsp.* (BCRC12910)	–	3.125	12.5
	*Vibrio parahaemolyticus*	< 0.4	–	–
	*Vibrio vulnificus* (YJ016)	–	3.125	12.5
	*Vibrio harveyi* (BCRC13812)	–	3.125	12.5
	*Vibrio harveyi* (BCRC12907)	–	6.25	12.5
	*Vibrio vulnificus*	12.5	6.25	12.5
	*Vibrio alginolyticus* (BCRC12829)	0.77	6.25	12.5
	*Pasteurella multocida*	0.77	–	–
	*Morganella morganii*	0.77	–	–
	*Aeromonas sobrio*	3.11	–	–
	*Aeromonas hydrophila*	3.11	–	–
	*Escherichia coli*	6.24	–	–
	*Pseudomonas aeruginosa*	–	> 100	60
	*Pseudomonas fluorescens*	200	–	–
	*Yersinia enterocolitica subsp.* (BCRC13999)	–	100	100
	*Flavobacterium meningosepticum*	< 0.4	–	–
	*Riemerella anatipestifer* (11 different strains)	–	–	6.25–50 (Average 27.8)
Other	*Trichomonas vaginalis* (ATCC 50143)			25–62.5
	*Trichomonas vaginalis*	–	–	12.5
	*Candida albicans*	25	–	25
	*Pichia pastoris*	495	–	–
	*Microsporosis canis*	50	–	–
	*Trichophytonsis mentagrophytes*	100	–	–
	*Cylindrocarpon sp.*	100	–	–

To evaluate the therapeutic effect of Epinecidin-1 on *Riemerella anatipestifer* infection, a study was carried out in 2009 [20]. This Gram-negative bacterium causes significant economic losses as it can infect ducks. Although an appropriate antibiotic treatment exists for this infection, antibiotic resistance is being increased [21, 22]. Investigation of Epinecidin-1 therapeutic effect on *R. anatipestifer* infected ducks as pretreatment, co-treatment, and post-treatment showed that the number of bacteria in duck greatly reduced in all three cases and the survival rate was significantly increased [20]. Study of two aquatic animals of tilapia (*Oreochromis mossambicus*) and grouper to reveal the therapeutic effect of Epinecidin-1 in *Vibrio vulnificus* infection showed that the simultaneous injection of bacteria and peptide significantly increased their survival. The half-life of this peptide in tilapia was 60–80 min following the injection [10]. In a study on *V. vulnificus* contaminated fish using zebrafish model system, it was shown that the pretreatment, co-treatment, and post-treatment with 1 μg of Epinecidin-1 could increase the survival rate of fish from 3 to 56.6%, 80, and 60% respectively [23].

Epinecidin-1 showed good bactericidal effects when exposed to sensitive and antibiotic-resistant *Pseudomonas aeruginosa*. The mouse model infected by multidrug-resistant *P. aeruginosa* strains also combated the infection more effectively with no adverse behavioral effects, hepatotoxicity, and nephrotoxicity when Epinecidin-1 applied [24, 25].

*Helicobacter pylori* is a gram-negative bacterium that causes active chronic gastritis and may cause complications such as malignancies, dyspepsia, and peptic ulcer [26]. The *H. pylori* infection is very important as the Nobel Prize in physiology in 2005 was awarded for the discovery of the peptic ulcer disease caused by an infection with *H. pylori* [27]. Following the development of antibiotic-resistant *H. pylori* strains, evaluation of AMPs has started for the treatment of this infection. Epinecidin-1 is one of the most potent AMPs which have been introduced so far [5]. This peptide can inhibit the growth of sensitive and antibiotic-resistant *H. pylori* strains with the MIC of 8–12 μg/ml. It has also shown that the oral administration of Epinecidin-1 (250 μg) could completely remove the *H. pylori* from the stomach in a C3H/HeN mice [28].

In a study performed in 2019 on mice, suffering from metronidazole-resistant trichomoniasis, it was reported that Epinecidin-1 could combat the infection effectively [29]. Trichomoniasis is a sexually transmitted disease caused by a protozoan called *Trichomonas vaginalis*. More than 140 million people worldwide are annually affected by this infection [30]. In two studies, the Epi-1 MIC has been reported as 12.5 μg/ml and 25–62.5 μg/ml for metronidazole-sensitive and -resistant strains of *T. vaginalis*, respectively [29, 31]. It has also shown that a 400 μg dose should be used for the complete elimination of infection in mice with trichomoniasis. A remarkable point of this report is that no damage was observed in vaginal epithelial cells of mice in 500 and 1000 μg doses. It can be attributed to the protective mucus layer on these cells [29].

### 4. Antiviral effect

Antiviral effects of many AMPs have been reported so far. However, according to the Antimicrobial Peptide Database (APD), antiviral effects have been evaluated and reported for only 185 out of more than 3000 registered AMPs. It should be noted that regarding the more difficult evaluation of antiviral properties, most of the AMPs have not been evaluated for these effects. Fortunately, Epinecidin-1 is among the AMPs with well investigated antiviral properties [3, 32].

Betanodavirus or nervous necrosis virus (NNV) is a virus classified in the Nodaviridae family. It is one of the main fish pathogens which causes much damage in cultured marine fishes each year [33]. Effect of Epinecidin-1 on this virus has been investigated in two studies. Wang et al. investigated the effect of Epinecidin-1 (1, 0.5, 0.1, and 0.05 μg) with pretreatment, post-treatment, and co-treatment on *E. coioides* infected by NNV and the best result was related to the co-treatment. Co-treatment of Epinecidin-1 and NNV through the anal canal could increase the survival rate of infected fish in a dose-dependent manner. For example, co-treatment of 1 μg/fish Epinecidin-1 ten minutes before the virus inoculation could increase the survival rate from 11 to 35% [34]. In another study, the maximal non-cytotoxic concentration of Epinecidin-1 to GF-1 and cBB cells was determined as 4 and 8 μg/ml, respectively. Therefore, the evaluation of Epinecidin-1 antiviral effects should be performed at lower concentrations. Investigation of Epinecidin-1 antiviral effect on nervous necrosis virus (NNV) in non-cytotoxic concentration for these two cell lines showed that this peptide was not able to completely block the virus infectivity [35], although it has been shown to be possible in higher concentrations [34].

The foot-and-mouth disease virus (FMDV) is a member of the picornavirus family and Aphthovirus genus that can cause foot-and-mouth disease in cloven-hoofed animals and is highly contagious. Although it has a low mortality rate, it can cause significant losses for animal husbandry in low milk and meat production as well as animal products [36]. In a study by Huang et al. in 2018 on the evaluation of the antiviral effects of five marine AMPs, it was observed that Epinecidin-1_22-42_ could combat FMVD more effectively. The BHK-21 cells were used in their study. The 50% cytotoxic concentration (CC50) of Epinecidin-1 for BHK-21 cells was 19.5 μg/ml and the 50% effective concentration (EC50) for viral inhibition was 0.6 μg/ml. Investigation of higher concentrations (10 × EC_90_ of Epinecidin-1) showed good virucidal activity against FMDV. It was also observed that the most effect of this peptide was during the viral adsorption stage [37].

Japanese encephalitis virus (JEV) is a flavivirus that causes encephalitic diseases in children [38]. It has shown that co-treatment of 1 μg/ml Epinecidin-1 plus JEV on BHK-21 cells, dropped the infection rate by 50% compared to the control. Also, a study on mouse model showed that with the co-injection of JEV and 200 μg/ml Epinecidin-1, all mice survived. However, the group of mice without Epinecidin-1 co-injection were died [39].

Evaluation of the gene expression of Epinecidin-1 under different conditions showed that the infection by Singapore grouper iridovirus (SGIV) up-regulated the Epinecidin-1 gene in *E. coioides* [40].

### 5. Anticancer effect

Most of the current cancer treatment methods are complex and have side effects. For example, chemotherapy causes serious side effects. Also, current anticancer drugs are often focused on the destruction of cells with a high rate of growth, and probably they are not able to differentiate between cancer and normal cells. In addition, the emergence of multidrug-resistant cancer cells which are capable of neutralizing the function of anticancer drugs is a new challenge. This happens through various mechanisms including repair of damaged DNA, transport of the drug out of the cell, and expression of drug detoxifying enzymes [41, 42]. Consequently, the focus of most of the studies was on the anticancer properties of various types of natural or chemical drug compounds. In recent years, with the increased research on AMPs, it has been observed that some of them also have anticancer effects. Epi-1 is one of the AMPs with reported anticancer properties in several studies [42, 43].

Anticancer AMPs are usually divided into two groups. The first group can kill cancer cells as well as normal mammalian cells. The second group is potent to kill cancer cells, while no activity against normal mammalian cells or with poor activity [44]. A study on the Epinecidin-1 cytotoxic effect on some normal and cancer cells showed that this peptide could effectively inhibit the growth of both tumor and normal cell lines at the concentrations above 2.5 μg/ml. Although the growth inhibition also occurred for normal cells, the rate of inhibition at the 2.5 μg/ml concentration was slightly lower for the normal cells (AML-12, NIH3T3, and WS-1) compared to the cancer cells (A549, HA59T/VGH, HeLa, HT1080, RAW264.7, and U937). It has also shown that the Epinecidin-1 induces cytotoxic effects and membrane lysis through the perturbation of the cancer cell membrane. Also, this peptide inhibits the necrosis in HT1080 cells (highly aggressive fibrosarcoma cell line) by downregulating the necrosis-related genes [45].

In another study, it was shown that Epinecidin-1 inhibited the proliferation and induced the apoptosis in human leukemia U937 cells. This seemed to occur through the increase in tumor necrosis factor (TNF)-α; interleukin (IL)-10, interferon (IFN)-γ, p53, IL-15, and IL-6. It was also observed that the ADP/ATP ratio which is an indication of mitochondrial dysfunction, was increased in these cells [46].

To date, there is not much research to clarify the exact mechanism of Epinecidin-1 anticancer function. However, it should be noted that the AMPs with a similar structure to Epinecidin-1 usually have the same anticancer mechanism [47]. Generally, a cancer cell is involved in the events that lead to an increase in the net negative charge of the cell surface. The cationic peptides are absorbed to this negatively charged cells, then the destruction of the cancer cell would occur. Although the normal cells usually have lower negative charges in the surface membrane, the cancer cells express many anionic molecules including phosphatidylserine (PS), sialic acid, membrane-associated glycoproteins, chaperone proteins HSP90 and GRP78 [42].

### 6. Wound healing effect

Epithelial cell proliferation and migration are two important factors in wound healing [48]. The effect of Epinecidin-1 has been recently evaluated on these factors using immortalized keratinocyte HaCaT cells. Epinecidin-1 did not show any cytotoxic effects on these cells at the concentration of 31.25 μg/ml. It is interesting that the treatment of cells with 15.625 μg/ml Epinecidin-1 resulted in increased cell numbers and proportions of actively dividing S-phase cells out of the total cells. So it can be concluded that the treatment with Epinecidin-1 promoted the cell proliferation and migration into the wounded region [49].

Furthermore, animal studies on MRSA-infected skin ulcers in mice and swine indicated that this peptide reduced the bacterial count in the wounded region, increased the angiogenesis, and enhanced wound closure [49, 50].

In a mouse model with MRSA-infected skin ulcer, use of Epinecidin-1 resulted in faster wound closure compared to the non-infected ulcers and infected but vancomycin-treated ulcers. Also, co-treatment with Epinecidin-1 and collagen enhanced the healing and re-epithelialization compared to Epinecidin-1 treatment alone. Evidence shows that Epinecidin-1 activity in MRSA-infected ulcers is not only related to the antimicrobial property, and this peptide also plays a role in the regulation of some cytokines. For example in MRSA-infected mice ulcers, treatment with Epinecidin-1 decreased serum levels of the proinflammatory cytokines TNF-α, IL-6, and MCP-1, and regulated the recruitment of monocytes and clearance of lymphocytes around the wounded region during healing [49].

In confirmation of previous findings, the results of another study on the effect of Epinecidin-1 on the healing of MRSA-infected swine burn ulcers showed that this peptide could accelerate the wound healing and provide protection against infection. Within 25 days, Epinecidin-1 increased epithelial activities, extracellular collagen compound formation, and vascularization. Also, treatment of swine with Epinecidin-1 suppressed the C-reactive protein (CRP) and the pro-inflammatory cytokine IL-6, while their expression induced in untreated swine group [50].

### 7. Mechanism of action

In this section, the discovered mechanisms of action of this peptide are introduced. In most studies, there are two main mechanisms involved to combat the infections, including (i) Direct attack on the cell membrane of pathogens (ii) Regulation of the immune system to combat infection.

#### Membrane attack

The main invasive mechanism of this peptide in exposure to pathogens is the destruction of the cell membrane [20, 31]. It has been observed that Epinecidin-1 reduces the negative surface charge following the binding to the bacterial surface. This peptide causes the saddle splay membrane curvature generation in bacterial membrane followed by pore formation, blebbing, budding, and vesicularization in membrane and lysis. Membrane lysis results in bacterial death [28, 46]. For example, the appearance of three pathogens of *Candida albicans*, *Propionibacterium acnes*, and *Trichomonas vaginalis* in exposure to Epinecidin-1 were evaluated in one study using scanning electron microscopy (SEM) and transmission electron microscopy (TEM). The comparison of their appearance before and after Epinecidin-1 treatment showed that this AMP caused breakage in the membrane and intracellular contents were transported outside the cell. *C. albicans* was also found to have a highly irregular shape, in addition to the membrane breaks. The morphological changes of Trichomonas were as membrane destruction and hollow appearance [31]. Another study evaluated the Epinecidin-1 effect on 11 strains of *R. anatipestifer* which is a pathogen of ducks, and it was shown that this peptide caused breaks in the membrane and the efflux of cellular contents [20]. Furthermore, observation of Epinecidin-1 effects on *P. aeruginosa* using TEM revealed membrane disruption and lighter electron density in the cytoplasm [25].

Fortunately, the toxic concentration threshold for human cells is much higher than the microbial cells. Epinecidin-1 at the concentration of 0.42 μM have no hemolytic effect on human RBCs. However, hemolytic effects appear to be highly dose-dependent at the 0.83 μM concentration [7, 29]. High doses of Epinecidin-1 did not exert toxic effects in oral, dermal, and eye irritation models [28].

#### Immune regulation

It has been shown in many studies that Epinecidin-1 combat infections through the regulation of the immune system. In Japanese encephalitis virus infection, Epinecidin-1 modulated the expressions of MCP-1, IL-6, IL-10, IFN-γ, TNF-α, and IL-12, and elevated the levels of anti-JEV-neutralizing antibodies in the serum [39].

It was also shown that Epinecidin-1 could play a role in the regulation of Mx2 and Mx3 genes expression [34, 35]. It should be noted that Mx genes exist in the genome of most vertebrates are responsible for a specific antiviral state against viruses [51]. In *V. vulnificus*-infected fish, Epinecidin-1 could also regulate immune response genes such as IL-10, IL-1b, TNF-α, and IFN-γ and help to control the infection by enhancing the immunity [23].

Co-injection of Epinecidin-1 and *P. aeruginosa* in a mouse model showed that this peptide was a good immunomodulator. Epinecidin-1 induced significant secretion of immunoglobulin G1 (IgG1) through the activation of Th2 cell response in infected mice. Treatment with this peptide during the septic shock also had a positive effect and probably involved protecting the liver against LPS-induced dysfunction and preventing damage to several other organs in septic mice [24].

In a study of immunostimulatory effect of Epinecidin-1 peptide in two models of grouper (*E. coioides*) and zebrafish (*Danio rerio*), it was shown that this peptide significantly increased the expression of some important genes of immunity such as (TNF)-1 in grouper and Toll-like receptor (TLR)4, IL-1b, nitric oxide synthase (NOS)2, and (NF)-κB in zebrafish [52].

According to these findings, researchers aimed to produce recombinant Artemia (a live feed for fry in aquaculture) to express Epinecidin-1. It was observed that supplementing adult or larva fish especially zebrafish with this transgenic Artemia strain, significantly increased the immunity against infections [53, 54].

In the study of mice peritonitis infection caused by *P. aeruginosa* ATCC 19660, it was shown that Epinecidin-1 decreased the concentrations of IL-1β, IL-6, and TNF-α. Interestingly, if the peritonitis caused by the resistant strains of *P. aeruginosa*, Epinecidin-1 treatment significantly increased plasma concentrations of IL-1β and TNF-α [25]. Epinecidin-1 in Raw264.7 macrophage cells could inhibit the inflammation through the LPS neutralization. It could also directly inhibit MyD88 via induction of the Smurf E3 ligase proteasome pathway. MyD88 is a protein that functions as an essential signal transducer in the IL-1 and TLR signaling pathways. These pathways regulate the activation of numerous proinflammatory genes. It is notable that LPS upregulates the MyD88 protein level [55, 56].

Epinecidin-1 also have been shown to combat *H. pylori* infection through immunologic changes in the host. For example, inhibited infection through in vivo depletion of CD4 + -FOXP3+ T Regulatory and Th17 subset populations, and aided in clearance of persistent *H. pylori* colonization. Flow cytometry and gene expression analysis of mouse splenic and gastric tissue indicated that Epinecidin-1 inhibits IL-10, and thereby affects FOXP3 expression levels and reduces pro-inflammatory cytokine responses [28].

In a duck model infected by *R. anatipestifer*, it was shown that Epinecidin-1 injection produced upregulated expression of the Mn superoxide dismutase (MnSOD) gene in brain tissue [20]. This gene plays an important role in host defense against the oxidative damage due to the infection-mediated inflammation [57].

### 8. Application of Epinecidin-1 in vaccine design

There is evidence of AMPs adjuvant properties that have made them suitable candidates for further studies. Adjuvant-like properties of AMPs have been reported for the Epinecidin-1 in several studies [58]. The co-treatment of Epinecidin-1 with *V. vulnificus* showed vaccine-like effects in addition to therapeutic effects. As the surviving zebrafish after 30 days of co-treatment were re-challenged with *V. vulnificus* and a survival rate of 45% was observed, while this rate was about 3% in the control group [23].

Similar properties have also been reported for the advantages of using Epinecidin-1 in vaccine production. As mentioned in section 4, co-injection of Epinecidin-1 with JEV virus prevented the death in mice. Interestingly, with the re-challenge of surviving mice of the previous step, all mice survived. While the unvaccinated control group did not survive. In the study of cytokines, it was found that Epinecidin-1 induced higher levels of Th2 cytokine than Th1 cytokine [39]. Similar results were obtained regarding the NNV-infected fish as well. When Epinecidin-1 treated fish were subjected to re-challenge with the co-treatment of Epinecidin-1 and NNV, the survival rate was even higher [34].

A report in 2012 showed that 30-day oral administration of Epinecidin-1 in two marine models of zebrafish and *E. coioides* exhibited excellent inhibition of bacterial growth against *V. vulnificus* [52].

### 9. Epinecidin-1 as a part of cleaning solutions

Cleaning solutions have broad applications in community hygiene. Usually, various antimicrobial agents are added to increase the microbicidal effects, such as pyrophosphate, zinc citrate, silicon oil, benzalkonium chloride, and cocamidopropyl betaine (CAPB). Unfortunately, it has been observed that the addition of these compounds could correlate with the emergence of resistant strains to antibiotics and disinfectants. Further research, therefore, is required to test new compounds [59, 60]. Epinecidin-1 is one of the few AMPs in which the efficiency has been evaluated as a part of the cleaning solution. It has shown that combination of this peptide with commercial cleaning solutions could significantly reduce the count of six pathogens including *Enterococcus faecalis*, *Escherichia coli*, *P. aeruginosa*, *S. aureus*, *Propionibacterium acnes*, and *C. albicans*, compared to the control. It has also been reported that parameters such as low temperature and pH have no significant effect on the Epinecidin-1 bactericidal activity in cleaning solutions. Therefore, it is possible to consider this AMP for combination with various disinfectants in the future [61].

## Discussion

The common property of all AMPs is to have lethal effects against one or more types of microbes (such as bacteria, virus, and protozoa). A factor that promotes the superiority of an AMP over others is a robust multi-functional activity. Regarding this issue, a strong bactericidal AMP with other activities such as immunomodulatory and anticancer features can be considered for research as an appropriate drug candidate [62]. In addition to antimicrobial activities of Epinecidin-1 on a wide range of microbes (bacteria, fungi, virus, and protozoa), other functions have been reported such as immunomodulatory, wound healing, and anticancer effects. LL-37 and Pexiganan are among the most known multi-functional AMPs that have been introduced so far. LL-37 is currently in Phase-II clinical trials for the treatment of Venous leg ulcers (VLUs) [63]. Pexiganan have also been evaluated clinically as a topical treatment for diabetic foot ulcer [64].

New and on-going findings associated with Epinecidin-1 encouraged us to write a comprehensive review of all previous studies on this peptide with high therapeutic potential to pave the way for further studies.

Epinecidin-1 is a cationic AMP with an alpha-helical structure. Similar to other cationic properties (structure and function), alpha-helical AMPs can absorb to the bacterial surface due to the positive charge. The structure and positive charge made this AMP very similar to LL-37 and Pexiganan [65].

Although many studies have been performed on this peptide, some characteristics are still not fully understood. The authors suggest the following arguments to be considered in future studies:

1. Investigation of exact anticancer mechanisms on a broader range of cancer cells.
2. Assess the possibility of producing topical drugs for the treatment of various infectious wounds, in particular, drug-resistant types.
3. This peptide has very powerful anti-*Helicobacter pylori* effects [34]. So, it can be considered for the treatment of such infections.
4. AMPs can have several functions, some of which have not been investigated for Epinecidin-1 yet. Therefore, evaluating other features such as antioxidant, anti-insect, protease inhibitor, spermicidal, and antitoxin effects can be considered.
5. A more comprehensive review of the toxicity and side effects of this peptide, especially on the intact animal.

## Conclusion

Epinecidin-1 is a strong multi-functional AMP with seven known functions including antibacterial, antifungal, antiviral, antiprotozoal, anticancer, immunomodulatory, and wound healing activities. This peptide has high potential for further research and application in new drugs.

## Abbreviations

AMP: Antimicrobial peptide
APD: Antimicrobial Peptide Database
CAPB: Cocamidopropyl betaine
CC50: 50% cytotoxic concentration
CRP: C-reactive protein
EC50: 50% effective concentration
ESTs: Expressed sequence tags
FMDV: Foot-and-mouth disease virus
IFN: Interferon
IL: Interleukin
JEV: Japanese encephalitis virus
LPS: Lipopolysaccharide
MIC: Minimum inhibitory concentration
MnSOD: Mn superoxide dismutase
MRSA: Methicillin-resistant *Staphylococcus aureus*
NNV: Nervous necrosis virus
NOS: Nitric oxide synthase
PS: Phosphatidylserine
SGIV: Singapore grouper iridovirus
TEM: Transmission electron microscopy
TLR: Toll-like receptor
TNF: Tumor necrosis factor
VLU: Venous leg ulcer
